# Supplementary material for: LTA4H extensively associates with mRNAs and lncRNAs indicative of its novel regulatory targets
Source: PeerJ. 2023 Mar 10;11:e14875. doi: 10.7717/peerj.14875 (PMC10010175; doi:10.7717/peerj.14875)
Supplement: File S2 [file peerj-11-14875-s005.zip › Raw data files/Motif_results/ablife/LTA4H_IP_2_vs_LTA4H_input_2/homerResults.html]

.// - Homer de novo Motif Results


# Homer *de novo* Motif Results (.//)

Known Motif Enrichment Results  
Gene Ontology Enrichment Results  
If Homer is having trouble matching a motif to a known motif, try copy/pasting the matrix file into
STAMP  
More information on motif finding results: HOMER
| Description of Results
| Tips
  
Total target sequences = 86438  
Total background sequences = 751313  
\* - possible false positive  

|  |  |  |  |  |  |  |  |  |
| --- | --- | --- | --- | --- | --- | --- | --- | --- |
| Rank | Motif | P-value | log P-pvalue | % of Targets | % of Background | STD(Bg STD) | Best Match/Details | Motif File |
| 1 |  | 1e-961 | -2.214e+03 | 39.65% | 28.49% | 156.0bp (159.2bp) | hsa-miR-4278 MIMAT0016910 Homo sapiens miR-4278 Targets (miRBase)(0.650) More Information \| Similar Motifs Found | motif file (matrix) |
| 2 |  | 1e-837 | -1.929e+03 | 26.36% | 17.37% | 149.4bp (185.2bp) | hsa-miR-4306 MIMAT0016858 Homo sapiens miR-4306 Targets (miRBase)(0.703) More Information \| Similar Motifs Found | motif file (matrix) |
| 3 |  | 1e-772 | -1.778e+03 | 43.99% | 33.65% | 161.4bp (166.4bp) | hsa-miR-3162-3p MIMAT0019213 Homo sapiens miR-3162-3p Targets (miRBase)(0.736) More Information \| Similar Motifs Found | motif file (matrix) |
| 4 |  | 1e-736 | -1.696e+03 | 29.94% | 21.01% | 156.1bp (164.7bp) | hsa-miR-3911 MIMAT0018185 Homo sapiens miR-3911 Targets (miRBase)(0.725) More Information \| Similar Motifs Found | motif file (matrix) |
| 5 |  | 1e-699 | -1.611e+03 | 61.45% | 51.33% | 155.7bp (155.2bp) | hsa-miR-938 MIMAT0004981 Homo sapiens miR-938 Targets (miRBase)(0.665) More Information \| Similar Motifs Found | motif file (matrix) |
| 6 |  | 1e-657 | -1.513e+03 | 56.60% | 46.75% | 157.8bp (146.7bp) | hsa-miR-3678-5p MIMAT0018102 Homo sapiens miR-3678-5p Targets (miRBase)(0.629) More Information \| Similar Motifs Found | motif file (matrix) |
| 7 |  | 1e-508 | -1.171e+03 | 64.62% | 56.11% | 154.8bp (167.7bp) | hsa-miR-516a-3p MIMAT0006778 Homo sapiens miR-516a-3p Targets (miRBase)(0.725) More Information \| Similar Motifs Found | motif file (matrix) |
| 8 |  | 1e-480 | -1.105e+03 | 26.03% | 19.11% | 163.0bp (170.7bp) | hsa-miR-1237 MIMAT0005592 Homo sapiens miR-1237 Targets (miRBase)(0.794) More Information \| Similar Motifs Found | motif file (matrix) |
| 9 |  | 1e-462 | -1.066e+03 | 70.89% | 63.05% | 156.5bp (159.3bp) | hsa-miR-4506 MIMAT0019042 Homo sapiens miR-4506 Targets (miRBase)(0.727) More Information \| Similar Motifs Found | motif file (matrix) |
| 10 |  | 1e-428 | -9.865e+02 | 73.26% | 65.88% | 159.0bp (149.7bp) | hsa-miR-720 MIMAT0005954 Homo sapiens miR-720 Targets (miRBase)(0.670) More Information \| Similar Motifs Found | motif file (matrix) |
| 11 |  | 1e-412 | -9.509e+02 | 58.42% | 50.64% | 157.3bp (159.2bp) | hsa-miR-3935 MIMAT0018350 Homo sapiens miR-3935 Targets (miRBase)(0.775) More Information \| Similar Motifs Found | motif file (matrix) |
| 12 |  | 1e-403 | -9.294e+02 | 71.13% | 63.85% | 152.5bp (156.4bp) | hsa-miR-3912 MIMAT0018186 Homo sapiens miR-3912 Targets (miRBase)(0.716) More Information \| Similar Motifs Found | motif file (matrix) |
| 13 |  | 1e-389 | -8.970e+02 | 39.31% | 32.09% | 156.2bp (146.0bp) | hsa-miR-4275 MIMAT0016905 Homo sapiens miR-4275 Targets (miRBase)(0.665) More Information \| Similar Motifs Found | motif file (matrix) |
| 14 |  | 1e-347 | -7.996e+02 | 46.03% | 38.97% | 162.4bp (156.7bp) | hsa-miR-1225-5p MIMAT0005572 Homo sapiens miR-1225-5p Targets (miRBase)(0.798) More Information \| Similar Motifs Found | motif file (matrix) |
| 15 |  | 1e-339 | -7.814e+02 | 81.58% | 75.71% | 152.7bp (148.4bp) | hsa-miR-4781-3p MIMAT0019943 Homo sapiens miR-4781-3p Targets (miRBase)(0.636) More Information \| Similar Motifs Found | motif file (matrix) |
| 16 |  | 1e-337 | -7.777e+02 | 68.09% | 61.31% | 149.6bp (159.8bp) | hsa-miR-548an MIMAT0019079 Homo sapiens miR-548an Targets (miRBase)(0.650) More Information \| Similar Motifs Found | motif file (matrix) |
| 17 |  | 1e-336 | -7.750e+02 | 58.01% | 50.98% | 160.0bp (145.8bp) | hsa-miR-4275 MIMAT0016905 Homo sapiens miR-4275 Targets (miRBase)(0.666) More Information \| Similar Motifs Found | motif file (matrix) |
| 18 |  | 1e-321 | -7.411e+02 | 22.30% | 16.91% | 161.0bp (148.8bp) | hsa-miR-4530 MIMAT0019069 Homo sapiens miR-4530 Targets (miRBase)(0.685) More Information \| Similar Motifs Found | motif file (matrix) |
| 19 |  | 1e-290 | -6.692e+02 | 24.68% | 19.32% | 158.2bp (151.9bp) | hsa-miR-3917 MIMAT0018191 Homo sapiens miR-3917 Targets (miRBase)(0.637) More Information \| Similar Motifs Found | motif file (matrix) |
| 20 |  | 1e-269 | -6.205e+02 | 2.94% | 1.26% | 135.7bp (162.7bp) | hsa-miR-4455 MIMAT0018977 Homo sapiens miR-4455 Targets (miRBase)(0.795) More Information \| Similar Motifs Found | motif file (matrix) |
| 21 |  | 1e-249 | -5.751e+02 | 48.71% | 42.67% | 159.9bp (150.1bp) | hsa-miR-646 MIMAT0003316 Homo sapiens miR-646 Targets (miRBase)(0.724) More Information \| Similar Motifs Found | motif file (matrix) |
| 22 |  | 1e-246 | -5.674e+02 | 73.66% | 68.15% | 156.3bp (142.0bp) | hsa-miR-4768-3p MIMAT0019921 Homo sapiens miR-4768-3p Targets (miRBase)(0.776) More Information \| Similar Motifs Found | motif file (matrix) |
| 23 |  | 1e-229 | -5.278e+02 | 44.49% | 38.78% | 156.3bp (154.4bp) | hsa-miR-3673 MIMAT0018096 Homo sapiens miR-3673 Targets (miRBase)(0.744) More Information \| Similar Motifs Found | motif file (matrix) |
| 24 |  | 1e-222 | -5.115e+02 | 25.55% | 20.78% | 158.6bp (149.2bp) | hsa-miR-4650-5p MIMAT0019713 Homo sapiens miR-4650-5p Targets (miRBase)(0.756) More Information \| Similar Motifs Found | motif file (matrix) |
| 25 |  | 1e-214 | -4.936e+02 | 90.02% | 86.33% | 152.1bp (159.0bp) | hsa-miR-548f MIMAT0005895 Homo sapiens miR-548f Targets (miRBase)(0.677) More Information \| Similar Motifs Found | motif file (matrix) |
| 26 |  | 1e-193 | -4.452e+02 | 55.48% | 50.15% | 161.9bp (153.8bp) | hsa-miR-4659a-5p MIMAT0019726 Homo sapiens miR-4659a-5p Targets (miRBase)(0.758) More Information \| Similar Motifs Found | motif file (matrix) |
| 27 |  | 1e-178 | -4.103e+02 | 68.25% | 63.38% | 148.3bp (158.2bp) | hsa-miR-495 MIMAT0002817 Homo sapiens miR-495 Targets (miRBase)(0.798) More Information \| Similar Motifs Found | motif file (matrix) |
| 28 |  | 1e-124 | -2.874e+02 | 2.00% | 1.01% | 129.9bp (144.8bp) | hsa-miR-508-3p MIMAT0002880 Homo sapiens miR-508-3p Targets (miRBase)(0.725) More Information \| Similar Motifs Found | motif file (matrix) |
| 29 |  | 1e-110 | -2.535e+02 | 54.19% | 50.19% | 161.7bp (150.7bp) | hsa-miR-3944-5p MIMAT0019231 Homo sapiens miR-3944-5p Targets (miRBase)(0.648) More Information \| Similar Motifs Found | motif file (matrix) |
| 30 |  | 1e-75 | -1.743e+02 | 49.92% | 46.60% | 170.7bp (144.3bp) | hsa-miR-634 MIMAT0003304 Homo sapiens miR-634 Targets (miRBase)(0.713) More Information \| Similar Motifs Found | motif file (matrix) |
